# Distinctive accuracy measurement of binary descriptors in mobile augmented reality

**DOI:** 10.1371/journal.pone.0207191

**Published:** 2019-01-03

**Authors:** Siok Yee Tan, Haslina Arshad, Azizi Abdullah

**Affiliations:** Center for Artificial Intelligence and Technology, Faculty of Information Science and Technology, Universiti Kebangsaan Malaysia, Bangi, Selangor, Malaysia; University of Science and Technology Beijing, CHINA

## Abstract

Mobile Augmented Reality (MAR) requires a descriptor that is robust to changes in viewing conditions in real time application. Many different descriptors had been proposed in the literature for example floating-point descriptors (SIFT and SURF) and binary descriptors (BRIEF, ORB, BRISK and FREAK). According to literature, floating-point descriptors are not suitable for real-time application because its operating speed does not satisfy real-time constraints. Binary descriptors have been developed with compact sizes and lower computation requirements. However, it is unclear which binary descriptors are more appropriate for MAR. Hence, a distinctive and efficient accuracy measurement of four state-of-the-art binary descriptors, namely, BRIEF, ORB, BRISK and FREAK were performed using the Mikolajczyk dataset and ALOI dataset to identify the most appropriate descriptor for MAR in terms of computation time and robustness to brightness, scale and rotation changes. The obtained results showed that FREAK is the most appropriate descriptor for MAR application as it able to produce an application that are efficient (shortest computation time) and robust towards scale, rotation and brightness changes.

## Introduction

Augmented Reality (AR) requires real-time tracking to trace a user’s or device’s position and register it with respect to the real world [1]. The ultimate goals of AR applications are to provide better management and ubiquitous access to information using seamless techniques in which the interactive real world is combined with an interactive computer-generated world, creating one coherent environment [2]. Briefly, AR involves integrating virtual objects into the real world. [2] defines an AR system as having three characteristics:

- Combined real and virtual objects in a real environment;
- Executed interactively and in real time; and
- Real and virtual objects registered (aligned) with each other.

Mobile devices such as smart phones have been recognized as one of the potential tools for AR [3–8]. Most of the smart phones nowadays provide a combination of a camera, accelerometer, GPS and other sensors making it as one of the most suitable device to provide computer vision application such as AR application [9]. Tracking from natural features is a complex problem and usually demands high computation power [10]. It is therefore difficult to use AR natural feature tracking in mobile device compared to personal computer (PC) platform because mobile devices have limited processing power, hardware and memory [11,12]. Hence, the selection of tracking algorithms needs to be given high attention in order to achieve optimum performance of AR in mobile platform.

The process of MAR is to identify and track natural features from the environment where local features from the scene image were matched with local features from the reference image. In order to carry out the matching process, the features’ keypoints from both scene and reference image must be detected and each detected keypoint must be described using the feature descriptor. Feature descriptors such as SIFT [13], SURF [14], BRIEF [15], ORB [16], BRISK [17] and FREAK [18] had been proposed as core components in image recognition, computer vision based tracking (visual tracking) and AR systems. Currently, researchers used these descriptors to develop the MAR application [19–24] without testing the performance of each descriptor used in the tracking process. Hence, it remains unclear which descriptors are appropriate for MAR application.

Previous researchers had worked to develop an efficient and robust MAR application [25–28]. Efficiency and robustness are the general performance measures of tracking. Efficiency is generally defining as the ability to track corresponding keypoints between consecutive frames in the shortest time possible. It is often interchanged with words such as “speed” and “fast”. Robustness can be defined as accurate tracking of corresponding keypoints between two frames in the presence of large changes in scale, rotation and brightness. “Accuracy” is another term often used to describe robustness of the tracking techniques [29]. Descriptor used in MAR application is the component that will directly affect the efficiency and robustness of an application [1,30]. In order to develop an optimum MAR application, a descriptor should able to act fast and at the same time robust to changes in viewing conditions, as well as tolerant to rotation and resistant to changes in brightness and uniform scaling.

Most recently some feature descriptors have been compared in PC AR application using Mikolajczyk dataset [30,31]. There are five descriptors involved in the comparison which is floating-point descriptor; SIFT and SURF, binary descriptor; FREAK and ORB and machine-learning descriptor; Ferns. The evaluation had been carried out to test the accuracy of descriptor in terms of scale and angle invariance but brightness invariance which is important for MAR application has not been evaluated. The computation time has also been evaluated but for both detection and description process without testing on computation time of description process separately. They conclude that Ferns and ORB yield the best performance in PC AR application [30].

To be precise on the scope of this paper, there is no need to address the floating-point descriptors, as floating-point descriptors are not suitable for mobile real-time application [32]. Hence, this paper will concentrate on binary descriptors performance for MAR application. This paper extended the evaluation of [30] by evaluating binary descriptor, namely BRIEF, ORB, BRISK and FREAK by using the same database [33] in mobile AR application and evaluate the computation time of description process itself without combining it with the detection process. This paper evaluated the accuracy of descriptor in term of brightness, scale and rotation invariance, which is important for MAR application.

## Background

**Fig 1:**
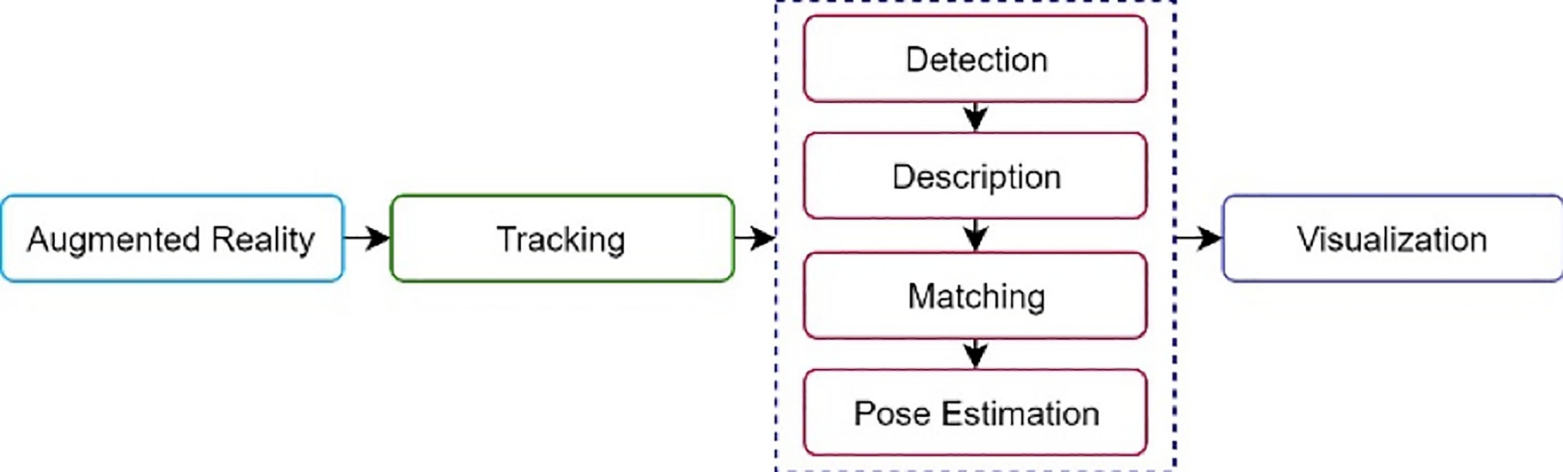
Tracking process in AR application.

AR requires real-time and accurate six degrees of freedom (6DoF) pose tracking of devices. Any particular AR application requires tracking technique to track the user’s or device’s position in order to register it in respect to the real world [34]. Such a tracking must run efficiently, typically required a total computation time less than 100 milliseconds [35]. Furthermore, it must be robust under many conditions such as varying brightness, scales and rotations [36–39]. Hence, the use of tracking algorithm in AR application is important in order to produce a high performance AR application. In AR, tracking is divided into four steps [40]. Basically, an image will be captured using phone’s camera and the image is converted into grey scale image. The first step in tracking process is detection, in which keypoint detectors were used to detect the natural features or keypoints of an image. The second step is to obtain a description of the image. A descriptor is required to describe or extract the keypoints detected in the detection process. Descriptors can be divided into two categories; floating-point descriptors and binary descriptors. SIFT and SURF are examples of floating-point descriptors, while BRIEF, ORB, BRISK and FREAK are examples of binary descriptors. The next step in the tracking process is matching. The keypoints of reference image should be stored in the database in advance to allow the system to match the points of an input image with those of reference image. Pose estimation is the last step in the tracking process. Pose estimation is performed to determine the position of a virtual object on top of the input image. After this process is completed, a 3D object can be superimposed on top of the detected image in the correct orientation [41]. Fig 1 shows the tracking process in AR application.

Descriptors of keypoints must be built to identify and match keypoints across images. The extraction process must be distinctive for each keypoint and need to be consistent under different viewpoints [42]. Existing feature descriptors will be briefly explained in this section, grouped by two categories; floating-point descriptors and binary descriptor.

### Floating-point descriptors

One of the most famous keypoint descriptors is SIFT (Scale Invariant Feature Transform) [43], which detects keypoints based on the Difference of Gaussians (DoG). Although SIFT was published in 1999, it still yields results that are competitive with state-of-the-art techniques. Apart from SIFT itself, several modified SIFT-like descriptors have been published, such as PCA-SIFT [44]. SURF (Speeded-Up Robust Feature) almost matched the quality of SIFT but accelerated the gradient computations using integral images [14]. To date, the SURF descriptor is considered as the most popular replacement for SIFT. Both SIFT and SURF have successfully demonstrated their high robustness and distinctiveness in a variety of computer vision applications [45,46]. However, the computation time required for floating-point descriptors is still too high for real-time applications, especially those running on limited computing power and memory capacity like smart phone [47]. Hence, binary descriptors aim to fill this gap.

### Binary descriptors

With the rapid growth of real-time applications, binary descriptors that achieved fast runtime and compact storage have become increasingly well-known [15]. They show similar quality as SIFT-like descriptors but at significantly lower computational costs and required small amounts of memory. The idea behind binary descriptors is that each bit in the descriptor is independent, and the Hamming distance can be used as a similarity measure instead of Euclidean distance. The four most recent and promising binary feature descriptors are BRIEF (Binary Robust Independent Elementary Feature) [15], ORB (Oriented Fast and Rotated BRIEF) [16], BRISK (Binary Robust Invariant Scalable Keypoints) [17] and FREAK (Fast Retina Keypoint) [18]. Hamming distance can be calculated effectively because the distance between descriptors were calculated by using XOR operation. Binary strings were generated by comparing the intensity of each pixel in the image. Binary string represents the area around the keypoint will be encoded in a string of “0” or “1”. Generally, single bits of a binary descriptor are calculated by comparing the intensity value of point ***x*** in a sampling pair with the intensity value of point ***y*** in the pair. A single bit of a binary descriptor ***B*** on patch ***p*** can be calculated using Eq 1.

$$B(p;x,y)≔\left\{ \begin{array}{l} \;1:\;I(p,x)<I(p,y)\\ \;0:\;otherwise \end{array},\right.$$ (1)

where *I*(*p*,*x*) is the pixel intensity at point *x* of a sampling pair and *I*(*p*,*y*) is the pixel int**e**nsity at point *y* of the sampling pair. A binary feature descriptor can be formed by concatenating the bits formed by *B*, as shown in Eq 2; where the *n* value for BRIEF and ORB is 256, while for BRISK and FREAK, it is 512.

$$\sum_{1\leq i\leq n}2^{i-1}\;B(p;x,y),$$ (2)

## Experimental setup

SIFT, SURF, ORB, FREAK and Ferns had been evaluated in a PC-based markerless AR and the computation times required by the detection, description and matching processes were compared [30]. However, they presented the computation time used by the detection process together with the description process and do not mention about number of keypoints involved in the process. The results showed that ORB required the shortest time to compute both the detection and description processes. They also tested the robustness of descriptors in terms of scale invariance, rotation invariance and occlusion. The Ferns descriptor obtained the highest matching rate compared to the other descriptors in all the robustness tests. Still, in all these evaluations, the computation times used by the descriptors were combined with the computation time used by the detector. Evaluating the computing time required by the descriptors themselves is important for determining the descriptors’ performances in AR applications. Moreover, they did not test the robustness of the descriptors in terms of brightness invariance which is important for AR applications [38]. Hence in this work we determined the distinctive and efficient accuracy measurements for several descriptors and identified which descriptor can function in the shortest amount of time and is robust to changes in terms of scale, rotation and brightness in markerless AR applications. This section will discuss the configuration of each test; computation time, rotation invariance, scale invariance and brightness invariance test, in an AR application using standard dataset. The testing was implemented on HTC One X+ android smart phone. It has built in camera and is able to record video with 1080 pixels at 28 fps or 720 pixels at 30 fps which fulfils the basic requirements for successful implementation of AR application. The source code for all these descriptors were obtained from OpenCV 2.4.9.

### Dataset

Although there are a vast number of datasets used to evaluate the performance of feature descriptors, the dataset used for this research was the well-known dataset introduced by [33] which had been used by most researchers [15,18,30]. The dataset consists of eight classes; bark, boat, bike, graffiti, wall, trees, leuven and ubc. Amsterdam Library of Object Images (ALOI) dataset [48] also had been used by most researchers [49,50]. The dataset consists of one-thousand small objects classes. This evaluation was carried out on a more specific task framework, similar to the one proposed by [51] and [18]. The images in this dataset are complement with extra rotation, scaling and brightness changes in this work to evaluate the performance of binary descriptors under various transformation and to isolate the effects of each transformation. The combination of detector and descriptor strongly affects the performances of the descriptors. Some descriptors are more discriminant for blobs than corners, but [18] noted that the global ranking of the matching performance remained the same regardless of the selected detector. Hence, the multi-scale AGAST detector introduced by BRISK was used throughout the tests [17].

### Evaluation metric 1: Efficiency

Efficiency is the ability to track corresponding keypoints between consecutive frames in the shortest time possible. Descriptor used in an MAR application should able to extract features in faster speed in order to create a MAR application that can act in real time. Hence, the first and most important measurement in this work was the computation time obtained by each descriptor. The detection and matching algorithm used throughout the measurement will be the same, which is BRISK detector and Brute Force Hamming Distance respectively. Eq 3 is used to measure the computation time used by descriptor. Let *M*_*m*_ denote the starting time, *M*_*t*_ denote the ending time, and *M*_*j*_ denote the total computation time. If *f* (*x*) is the function of each process (image capture, grey scale converter, keypoint detection, keypoint description, matching, pose estimation and visualization), then the computation time for each process is defined as:

$$f(x)=M_{j}(M_{t}-M_{m})$$ (3)

### Evaluation metric 2: Robustness

Robustness in various changes is the general performance measurement or a requirement for MAR application. Robustness can be defined as accurate tracking of corresponding keypoints between reference image and input image in the presence of large changes in scale, rotation and brightness. The evaluation criterion of robustness is based on the number of correct matches and the total number of matches obtained from reference image and input image. Two region A and B from reference image and input image respectively are matched if the distance *d* between their descriptor *D*_*A*_ and *D*_*B*_ is below a threshold. Each descriptor from the reference image is compared with each descriptor from the transformed input image and obtained the number of correct matches. Hence, accuracy of descriptor is the number of correctly matched regions with respect to total number of matches between reference image and input image of the same scene. Eq 4 is used in all the robustness evaluation includes rotation invariance, scale invariance and brightness invariance.

$$Accuracy\;(\%)=\frac{Number\;of\;Correct\;Matches}{Number\;of\;Matches}x\;100$$ (4)

#### Rotation invariance

**Fig 2:**
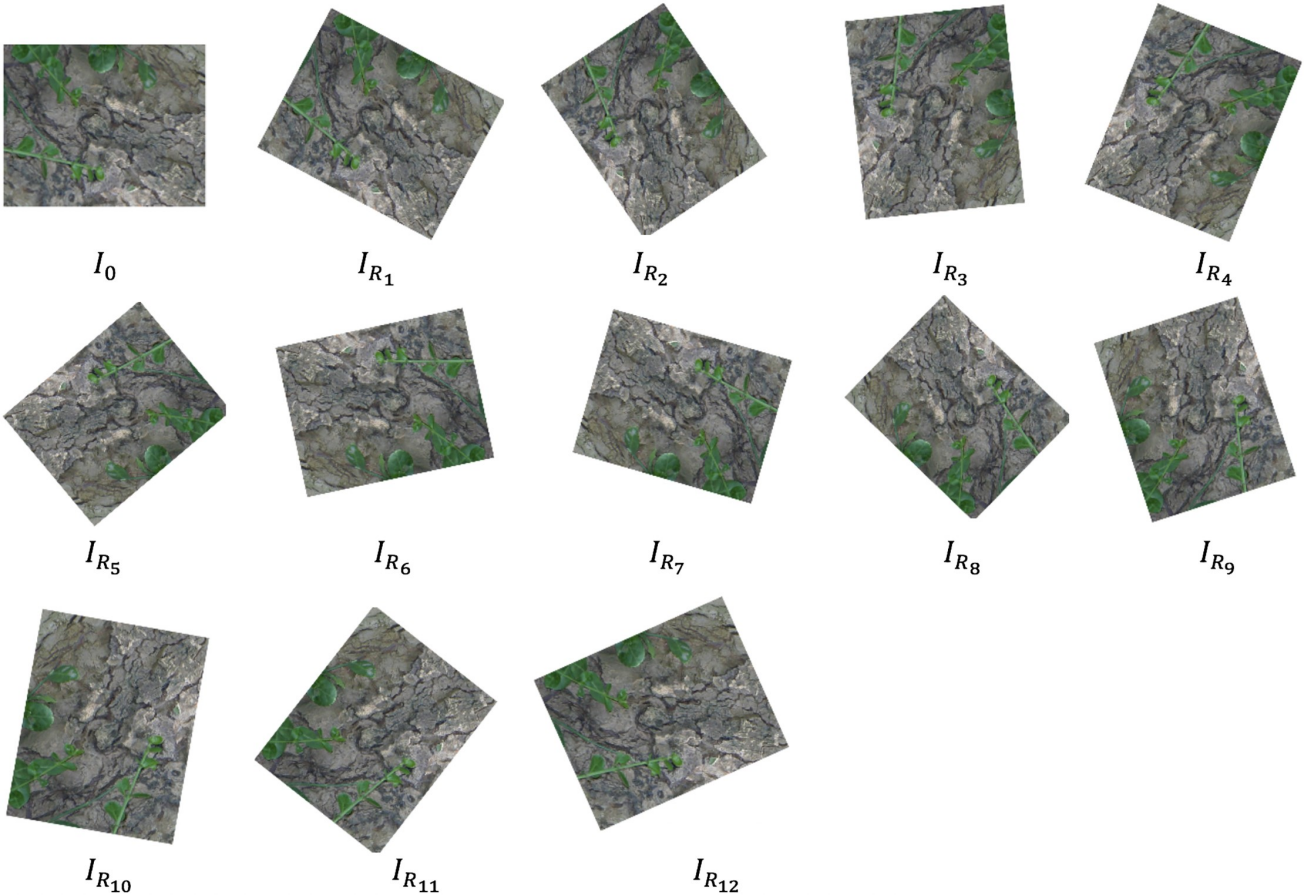
Rotation transformation using bark image.

Robustness of descriptors in terms of rotation changes are evaluated using leuven, bot and bark images from Mikolajczyk dataset and christmas bear, lab-keys and apricot images from ALOI dataset. The rotation invariance test applied affine rotation for both images around the center. The images were rotated by measuring the angle from the center. Let *I*_*o*_ be the original image and *I*_*R*_ be the rotated image. A total of 11 rotated images were produced and each rotated image can be defined using Eqs 5–7. Fig 2 shows the example of rotation transformation using bark image.

$$I_{o}=0\;degree$$ (5)

$$\begin{array}{c} I_{R_{1}}=(I_{o},28\;degrees)\\ \vdots \end{array}$$ (6)

$$I_{R_{12}}=(I_{R_{11}},28\;degrees)$$ (7)

#### Scale invariance

**Fig 3:**
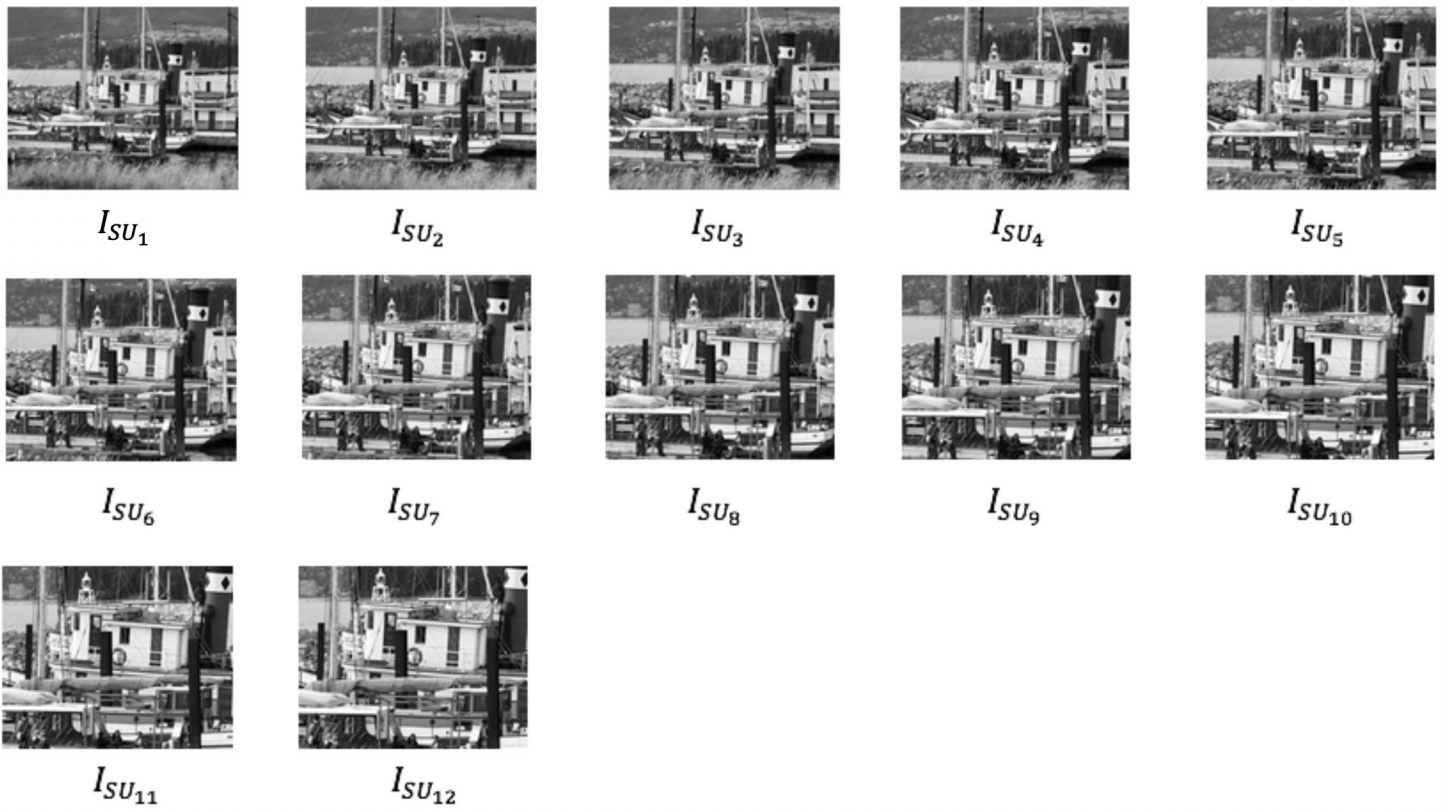
Scaled up transformation using boat image.

Leuven, bot and bark images from Mikolajczyk dataset and christmas bear, lab-keys and apricot images from ALOI dataset were used in scale invariance test. The scaling operation was applied to the original image. Let *I*_*o*_ be the original image and *I*_*SU*_ denote the scaled up or zoomed in images. A total of 12 scaled up images were produced and each scaling up image was performed based on Eqs 8–10. Fig 3 shows the example of scaled up transformation using boat image.

$$I_{{SU}_{1}}=I_{o}+5\%$$ (8)

$$\begin{array}{c} I_{{SU}_{2}}=I_{{SU}_{1}}+10\%\\ \vdots \end{array}$$ (9)

$$I_{{SU}_{12}}=I_{{SU}_{11}}+10\%$$ (10)

**Fig 4:**
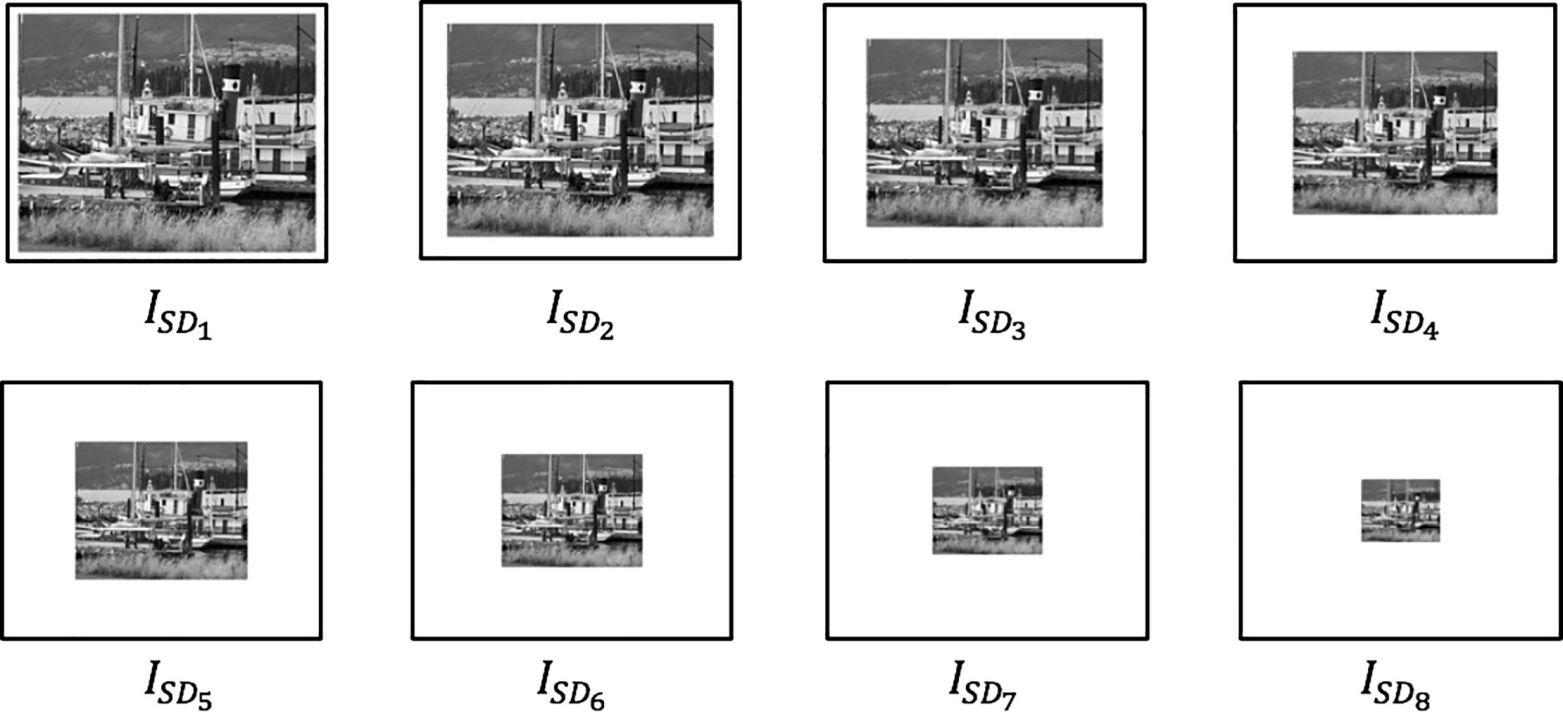
Scaled down transformation using boat image.

Let *I*_*SD*_ denote the scaled down images. A total of 8 scaled down images were produced and each scaled down or zoomed out image can be defined using Eqs 11–13. Fig 4 shows the example of scaled down transformation using boat image.

$$I_{{SD}_{1}}=I_{o}-5\%$$ (11)

$$\begin{array}{c} I_{{SD}_{2}}=I_{{SD}_{1}}-10\%\\ \vdots \end{array}$$ (12)

$$I_{{SD}_{8}}=I_{{SD}_{7}}-10\%$$ (13)

#### Brightness invariance

**Fig 5:**
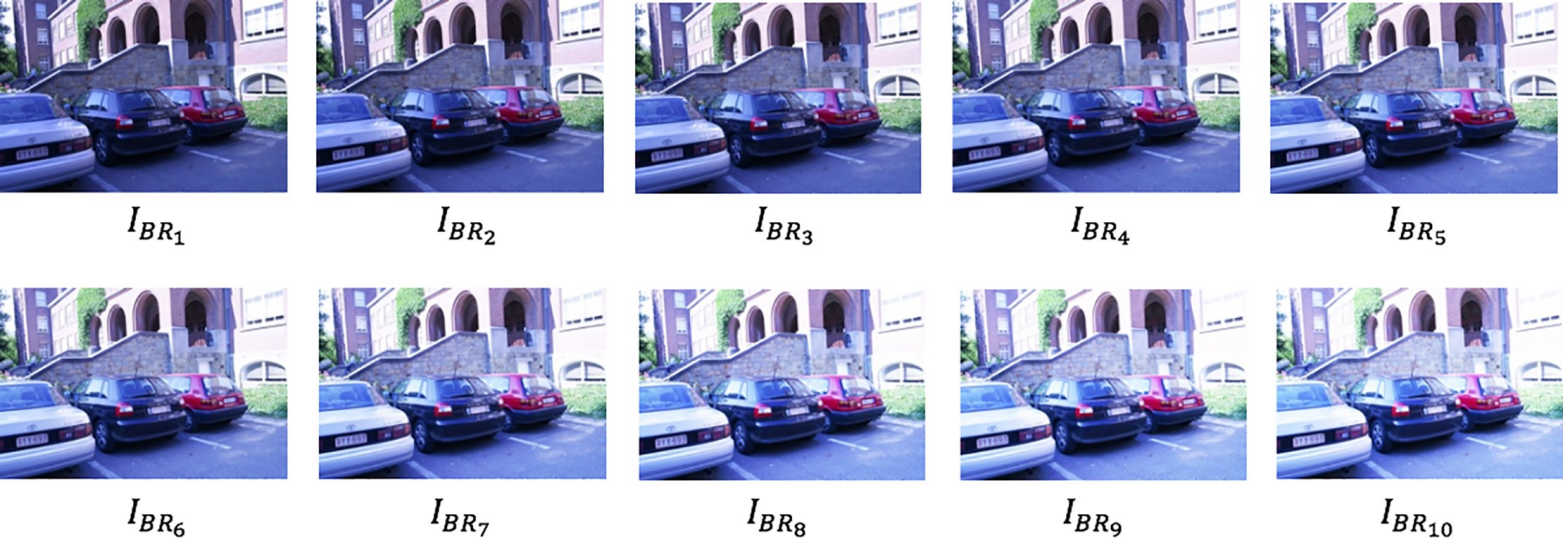
Brightness transformation (higher brightness value) using leuven image.

Leuven, bot and bark images from Mikolajczyk dataset and christmas bear, lab-keys and apricot images from ALOI dataset were used in brightness invariance test. The brightness value changes were applied to the original image. Let *I*_*o*_ be the original image and *I*_*BR*_ denote the brighter images (higher brightness value). A total of 10 brighter images were produced and performed based on Eqs 14–16; where R, G and B denote Red, Green and Blue, respectively, as the brightness value changes was applied to RGB images. Example of the brightness transformation (higher brightness value) using leuven images are shown in Fig 5.

$$I_{{BR}_{1}}={(I}_{o}•R\;x\;15)+{(I}_{o}•G\;x\;15)+{(I}_{o}•B\;x\;15)$$ (14)

$$\begin{array}{c} I_{{BR}_{2}}={(I}_{{BR}_{1}}•R\;x\;15)+{(I}_{{BR}_{1}}•G\;x\;15)+(I_{{BR}_{1}}•B\;x\;15)\\ \vdots \end{array}$$ (15)

$$I_{{BR}_{10}}={(I}_{{BR}_{9}}•R\;x\;15)+{(I}_{{BR}_{9}}•G\;x\;15)+(I_{{BR}_{9}}•B\;x\;15)$$ (16)

**Fig 6:**
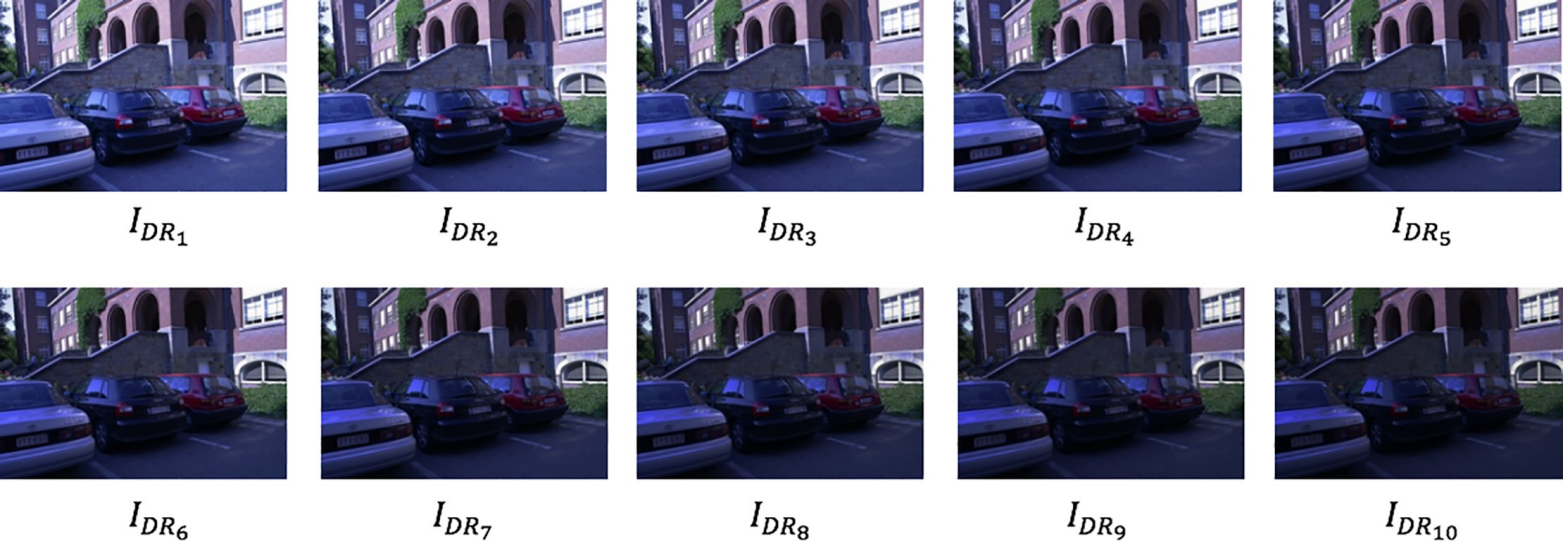
Brightness transformation (lower brightness value) using leuven image.

Similarly, let *I*_*DR*_ denote the darker images (lower brightness value). A total of 10 darker images were produced and performed based on Eqs 17–19; where R, G and B denote Red, Green and Blue, respectively, as the brightness value changes was applied to RGB images. Fig 6 shows the example of brightness transformation (lower brightness value) using leuven image.

$$I_{{DR}_{1}}={[I}_{o}•R\;x\;(-15)]+{[I}_{o}•G\;x\;(-15)]+{[I}_{o}•B\;x\;(-15)]$$ (17)

$$\begin{array}{c} I_{{DR}_{2}}={[I}_{{DR}_{1}}•R\;x\;(-15)]+[I_{{DR}_{1}}•G\;x\;(-15)]+[I_{{DR}_{1}}•B\;x\;(-15)]\\ \vdots \end{array}$$ (18)

$$I_{{DR}_{10}}={[I}_{{DR}_{9}}•R\;x\;(-15)]+[I_{{DR}_{9}}•G\;x\;(-15)]+[I_{{DR}_{9}}•B\;x\;(-15)]$$ (19)

## Results and discussion

This section discussed the experimental results of the evaluation. The performance of every binary descriptor; BRIEF, ORB, BRISK and FREAK is compared in terms of computation time, rotation, scale invariance and brightness invariances.

### Computation time

In order to measure the computation time of description process, the algorithm used to perform detection and matching process is the same. BRISK and Hamming Distance were used as detector and matching algorithm respectively, whereas, the description of each process is using BRIEF, ORB, BRISK and FREAK to test the exact computation time. The computation time of each process is calculated by using Eq 3. Computation time used to carry out keypoint detection, description and matching process is recorded for 50 times and every 500 keypoints. The results shown in Table 1 are the average computation time used to perform each process and the total computation time of the entire tracking process.

**Table 1:** Comparison of computation time for each process.

Descriptor	BRIEF Time (ms)	ORB Time (ms)	BRISK Time (ms)	FREAK Time (ms)
Capture Image	1.3	1.3	1.3	1.3
Convert to Grey Scale Image	2.4	2.3	2.3	2.3
Detection (500 keypoints)	14.1	14.5	14.3	13.8
**Description** **(500 keypoints)**	**7.5**	**9.4**	**5.2**	**4.3**
Matching (500 keypoints)	1.9	2.3	2.2	1.9
Pose Estimation	4.5	4.9	4.6	4.1
Visualization	1.9	2.0	1.7	1.4
Total Computation Time	33.6	36.7	31.6	29.1

The evaluation showed that the total computation time of tracking process using FREAK as a descriptor obtained the best result; 29.1ms. Conversely, the computation time using ORB descriptor obtained the longest computation time of 36.7ms. All these evaluations are showing that the tracking process using binary descriptor can work in real-time in mobile because the computation time are less than 100ms.

Computation time used for each process; captured image with camera, converted to grey scale image, keypoint detection, keypoint matching, pose estimation and visualization using different descriptors are approximately the same. For example, the detection process using BRISK algorithm to detect 500 keypoints in the tracking process using BRIEF, ORB, BRISK and FREAK descriptors are 14.1ms, 14.5ms, 14.3ms and 13.8ms, respectively. This is because the algorithm used for each detection process are the same.

Computation time used to describe 500 keypoints using different feature descriptors has a huge difference between each other. Computation time used by FREAK descriptor has secured the shortest time of 4.3ms, while the computation time used by BRISK and BRIEF are 5.2ms and 7.5ms, respectively. Computation time used by ORB descriptor to describe 500 keypoints are the longest; 9.4ms. Hence, FREAK descriptor is the most efficient descriptor as it can function in shortest computation time for mobile AR application compared to other binary descriptors.

### Rotation invariance

**Fig 7:**
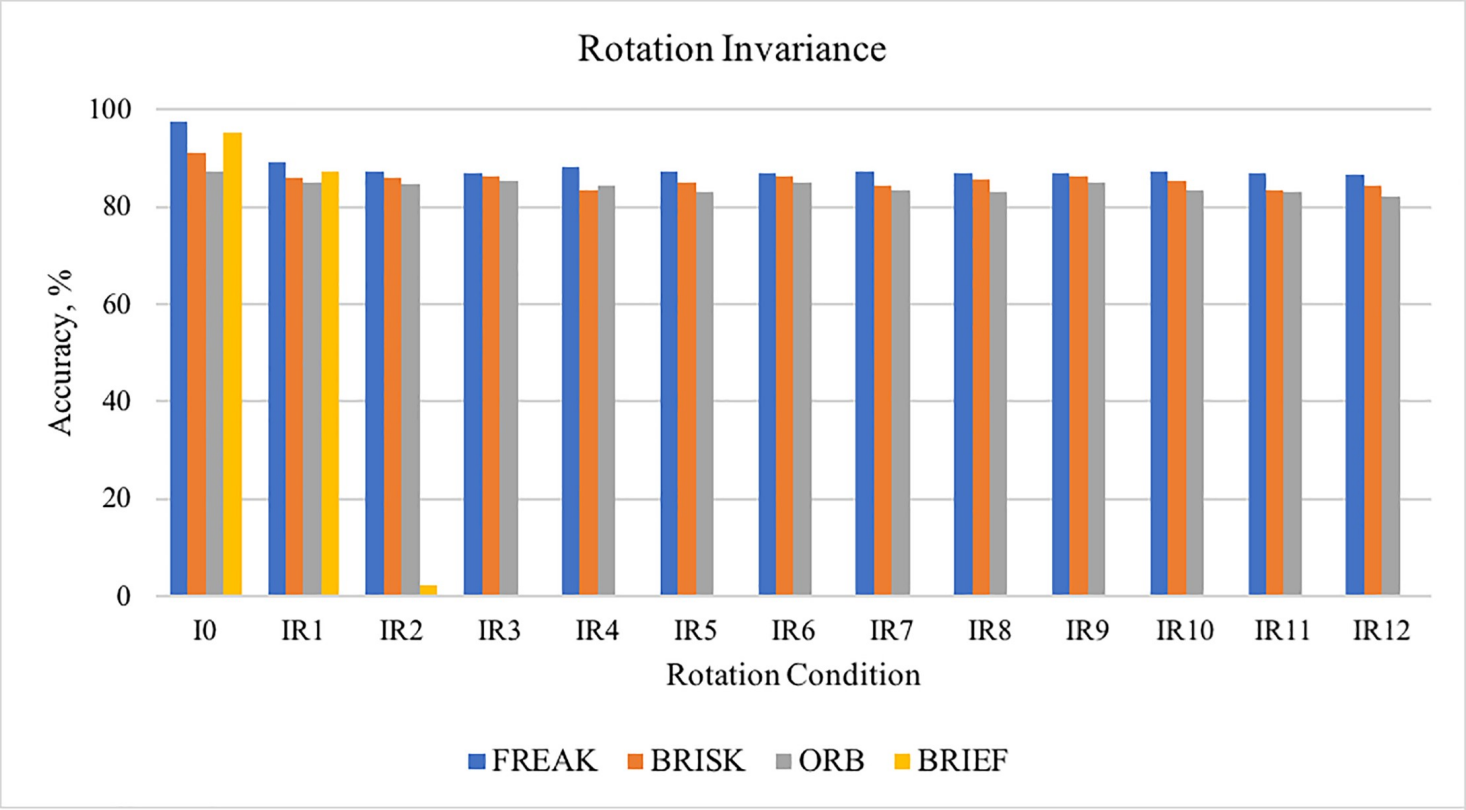
Accuracy obtained by each descriptor in different rotation conditions.

A total of 52 testing were carried out to evaluate the robustness of descriptor in rotation, which is 4 *descriptors x* 13 *sequence images*. The images were rotated at 28 degrees at the center sequentially. The number of correct matches and the number of matches were recorded in order to calculate the percentage of accuracy. This testing uses six images; leuven, boat and bark from Mikolajczyk dataset and christmas bear, lab-keys and apricot from ALOI dataset, therefore, each image that have the same rotation conditions were repeated 150 times (6 *images* × 25 *times*). For example, the boat image with $I_{R_{2}}$ condition was tested repeatedly for 25 times and the rest of the images; leuven, bark, lab-keys and apricot with $I_{R_{2}}$ condition was also tested repeatedly for 25 times each. Fig 7 summarize the robustness of each descriptor in various rotated images.

The results clearly showed that all the descriptors were most accurate at condition *I*_0_ as the image is the original image without any rotation changes. The highest percentage of accuracy at condition *I*_0_ was obtained by FREAK descriptor, 97.54% and followed by BRIEF descriptor, 95.22%, BRISK descriptor, 91.23% and ORB descriptor, 87.40%. Throughout the complete sequence of rotated images, from I_o_ to $I_{R_{12}}$, the accuracy did not change abruptly when using FREAK, ORB and BRISK, but the accuracy percentage of BRIEF dropped dramatically after $I_{R_{1}}$. The accuracy percentage of BRIEF descriptor drops to 0% after the image rotated at condition $I_{R_{3}}$. Therefore, BRIEF descriptor is not suitable for mobile AR application because BRIEF descriptor is not designed to extract features that have high rotation variation. This work also analysed the result using One Way Anova and mean. The results show that FREAK achieved the highest mean percentage of accuracy (88.135%) followed by BRISK (85.705%) and ORB (84.299%) while BRIEF obtained the lowest percentage of accuracy (14.211%). BRIEF descriptor also showed a significant difference compared to other descriptors in One Way Anova test. FREAK, BRISK and ORB are robust to rotation invariance and obtained a high mean percentage of accuracy in rotation variation test, but referring to Fig 7 and the mean test, FREAK slightly outperformed the other descriptors.

### Scale invariance

**Fig 8:**
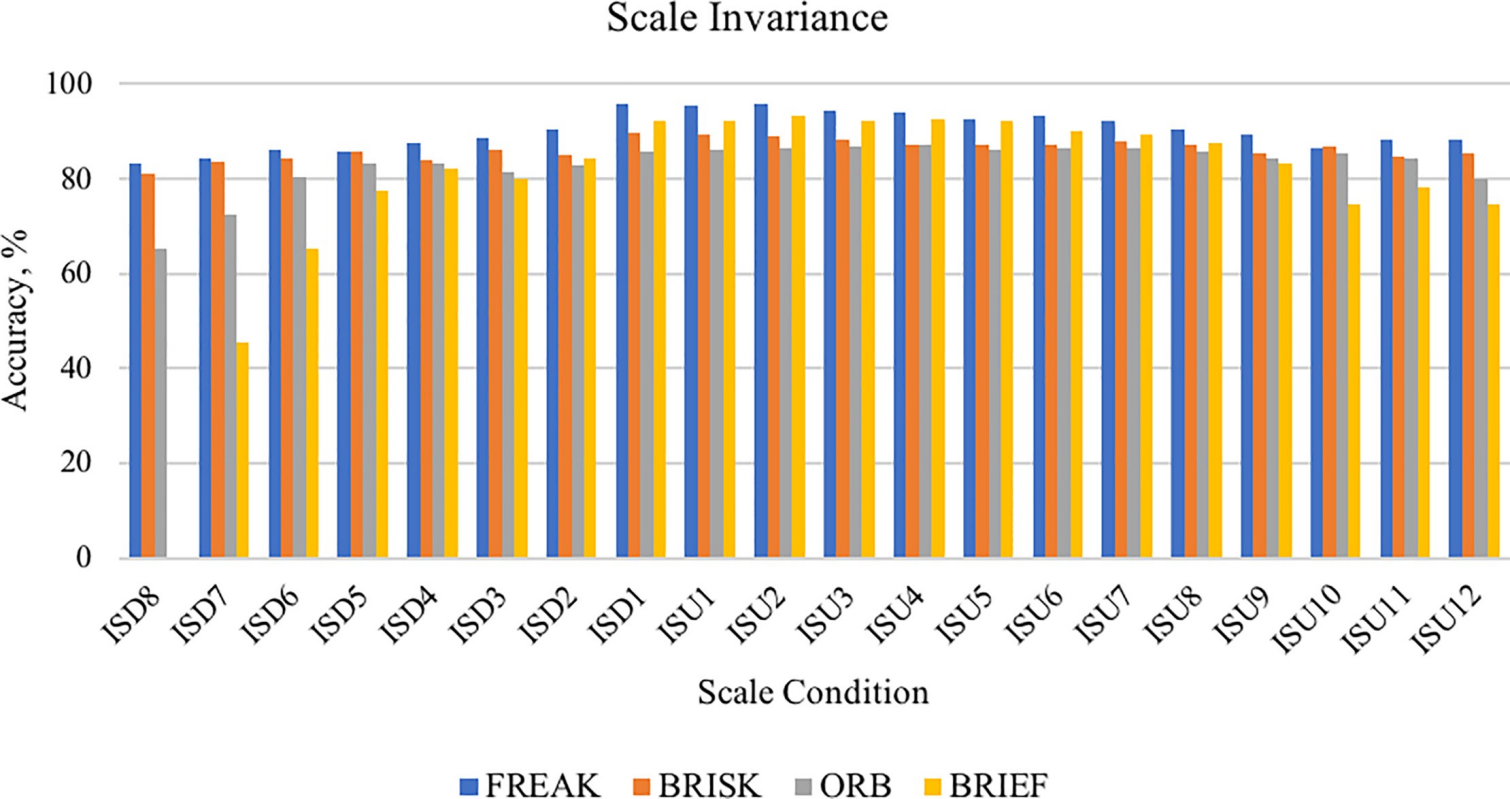
Accuracy obtained by each descriptor in different scale conditions.

Robustness of descriptors in terms of scale invariance were tested using leuven, boat and bark images from Mikolajczyk dataset and and christmas bear, lab-keys and apricot images from ALOI dataset. The configuration of the testing is similar to the testing in rotation invariance. A total of 80 testing were carried out to evaluate the robustness of descriptor in scale variation, which is 4 *descriptors x* 20 *sequence images*. This testing uses six images, therefore each image that have the same rotation conditions were repeated 150 times (6 *images* × 25 *times*). Fig 8 showed the robustness of each descriptor in various scale images.

The results show that the descriptors obtain their highest accuracy percentage at scale conditions $I_{{SD}_{1}},\;I_{{SU}_{1}}$ and $I_{{SU}_{2}}$. This is because the three images had undergone a minimum scale changes and most of the descriptors able to function accurately. As the camera starts to zoom out ($I_{{SD}_{1}}-I_{{SD}_{8}}$) or moving away from the input image, the accuracy percentage of each descriptors began to decline. FREAK, BRISK and ORB descriptor are still able to function by extracting features and continuing the matching process. Percentage of accuracy obtained by FREAK, BRISK and ORB at scale condition $I_{{SD}_{8}}$ are 83.20%, 81.21% and 65.33%, respectively. However, BRIEF descriptor obtained 0% accuracy because there are insufficient features to perform the matching process. Hence, BRIEF descriptor is not suitable for mobile AR application because BRIEF is not robust to scale invariance. Percentage of accuracy of each descriptor was analysed using One Way Anova and mean to identify the most robust descriptor in scale variation. The results here shown that FREAK obtained the highest mean percentage of accuracy (90.156%) followed by BRISK (86.246%), ORB (82.986%) and BRIEF (78.391%). Therefore, researchers concluded that FREAK, BRISK and ORB are descriptors that function robustly for scale variation in mobile AR application compared to BRIEF descriptor. However, FREAK again achieved the highest accuracy percentage among the others and hence indicated as the most robust descriptor in scale variation test.

### Brightness invariance

**Fig 9:**
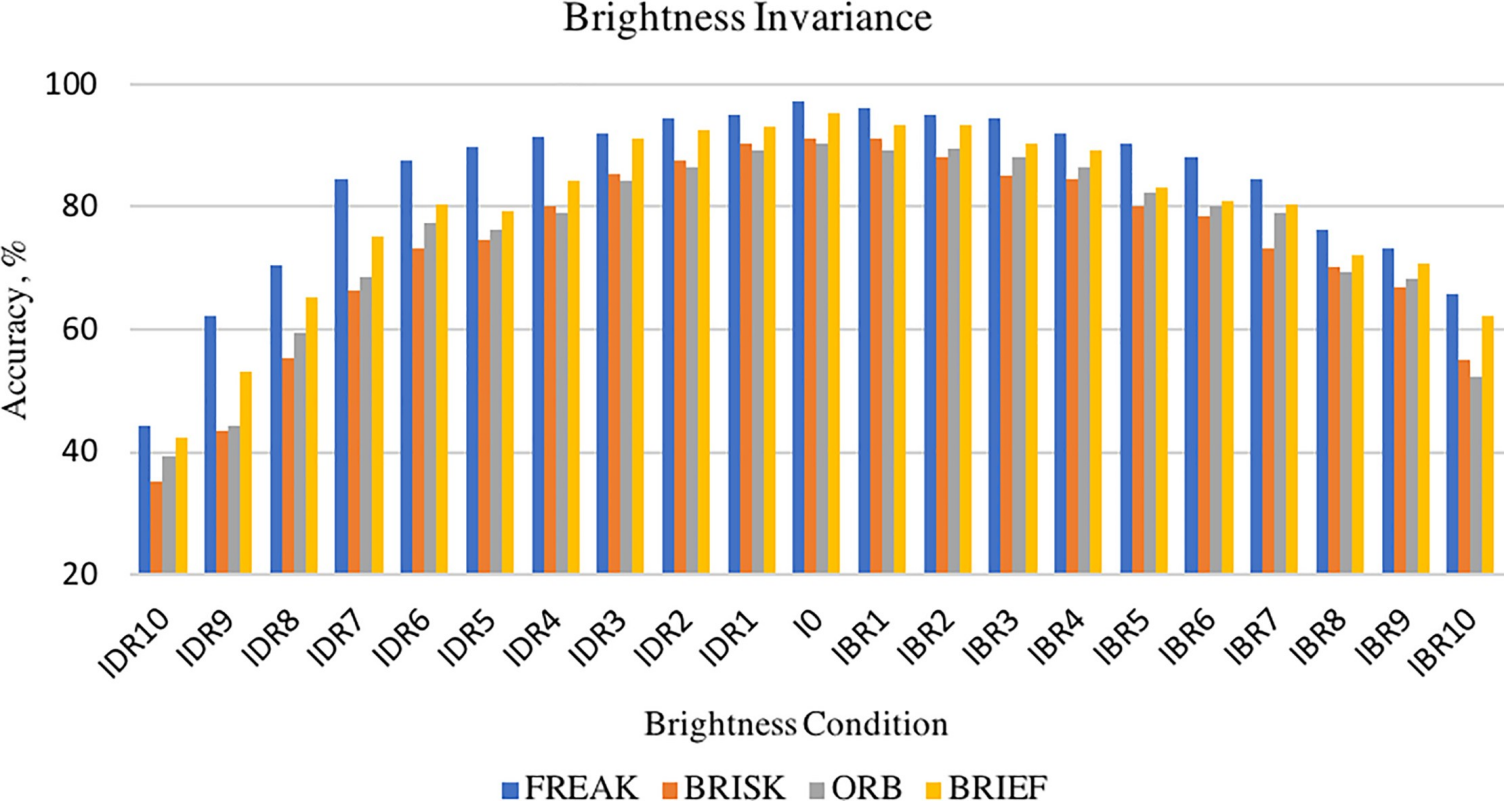
Accuracy obtained by each descriptor in different brightness conditions.

Robustness of descriptors in terms of brightness invariance were tested using leuven, bot and bark images from Mikolajczyk dataset and and christmas bear, lab-keys and apricot images from ALOI dataset. A total of 84 testing (4 *descriptors x* 21 *sequence images*) were carried out to evaluate the robustness of descriptor in different brightness condition. The number of correct matches and the number of matches obtained from each test were recorded in order to calculate the percentage of accuracy. Each image that have the same brightness conditions were tested repeated 150 times (6 *images* × 25 *times*). For example, the chrismas bear image with $I_{{DR}_{2}}$ condition was tested repeatedly for 25 times and the rest of the image; leuven, boat, bark, lab-keys and apricot with $I_{{DR}_{2}}$ conditions was also tested repeatedly for 25 times each. Fig 9 shows the accuracy percentage obtained by each descriptor in the presence of brightness changes.

The results showed that the highest accuracy percentages were obtained when the brightness conditions are at $I_{{DR}_{1}}$ and $I_{{BR}_{1}}$. This is because of these two brightness conditions had undergone a minimum change in brightness value which allows the descriptors to function robustly. When the brightness value decreases (condition $I_{{DR}_{10}}‑I_{{DR}_{1}}$) or increases (condition $I_{{BR}_{10}}‑I_{{BR}_{1}}$), the number of correct matches obtained by all the descriptors decreases dramatically. For example, accuracy of FREAK descriptor obtained at brightness condition $I_{{DR}_{1}}$ is 94.95% but when the brightness value decreases to condition $I_{{DR}_{10}}$, the accuracy obtained by FREAK descriptor is also decreased to 44.33%. Similarly, at the brightness condition $I_{{BR}_{1}}$, FREAK descriptor obtained 96.23% accuracy, but when the brightness value increases to condition $I_{{BR}_{10}}$, the accuracy decreased to 65.90%. However, the matching process does not fail in any of the conditions although the accuracy percentage decreased when brightness value increases or decreases. The percentage of accuracy of each descriptor was analysed using One Way Anova and mean to identify the most robust descriptor in brightness variation. The results showed that FREAK obtained the highest mean percentage of accuracy (84.041%) followed by BRIEF (79.410%), ORB (75.195%) and BRISK (74.057%). FREAK descriptor yields to be the most robust descriptor in brightness invariance test because the accuracy decline rate is less compared to other descriptors.

### Overall performance

Performance ranking for FREAK, BRISK, ORB and BRIEF in computation time, scale invariance, rotation invariance and brightness invariance are summarized in Table 2. The value of performance ranking is given based on the mean of each testing. The descriptor that obtained the lowest mean in computation time test was labelled as “1” (best) and the descriptor that obtained the highest mean was labelled as “4” (worst) in the performance ranking. Whereas in the robustness testing, the descriptor that obtained the highest mean was labelled as “1” (best) and the descriptor that obtained the lowest mean was labelled as “4” (worst).

**Table 2:** Performance ranking for each descriptor.

	Computation Time	Scale	Rotation	Brightness	Overall Performance	Performance Ranking
FREAK	1.0	1.0	1.0	1.0	1.0	1
BRISK	2.0	2.0	2.0	4.0	2.5	2
ORB	4.0	3.0	3.0	3.0	3.25	3.5
BRIEF	3.0	4.0	4.0	2.0	3.25	3.5

Based on Table 2, FREAK descriptor obtained the highest position in the performance ranking followed by BRISK descriptor (second position), ORB and BRIEF descriptor were both at 3.5 position, FREAK descriptor achieved the best performance compared to others descriptor in all the testing include efficiency and robustness test. Therefore, FREAK descriptor had been identified as the most appropriate descriptor for mobile AR application.

FREAK descriptors able to perform efficiently (low computation time) due to the sampling pairs structure of FREAK descriptor is using a coarse-to-fine apporach which matches with the model of human retina. FREAK takes advantage of this coarse-to-fine structure to further speed up the extraction using a cascade approach. FREAK descriptor first compare only the first 128 bits which representing coarse information during matching the two features. If the distance of the two features is smaller than a threshold, FREAK descriptor only further continue the comparison with the next 128 bits to analize finer information. As a result, a cascade of comparisons is performed accelerating even further the matching as more than 90% of the features are discarded with the first 128 bits of FREAK descriptor.

FREAK is a binary descriptor that is similar to BRIEF, ORB and BRISK, but with the added advantages of ratation invariance and learned sampling pairs which biologically inspired by the retinal pattern in the eye. FREAK descriptor suggests to use the retinal sampling grid which is also circular with the difference of having higher density of points near the center. The density of points drops exponentially. Each sampling point is smoothed with a Gaussian kernel where the radius of the circle illustrates the size of the standard deviation of the kernel. FREAK improves upon the sampling pattern and method of pair selection that BRISK descriptor uses. BRISK select pairs according to their spatial distance, however the selected pair can be highly correlated and not discriminat. Consequently, FREAK learn the pairs by maximizing variance of the pairs and taking pairs that are not correlated. This had lead to a more accurate description of the keypoints and make FREAK descriptor able to perform robustly under various changes.

## Conclusion

This research paper presents a distinctive and efficient method for measuring the accuracy of binary descriptors for mobile AR applications using Mikolajczyk dataset and ALOI dataset. Comparative accuracy tests were performed for FREAK, BRISK, ORB and BRIEF descriptors to determine the most appropriate descriptor (efficiency and robustness) in mobile AR applications. Based on the accuracy measurement results, FREAK is recommended as the best binary descriptor for mobile AR applications, yielding the fastest computation time of all the descriptors. Furthermore, FREAK achieved good results in rotation invariance, scale invariance and brightness invariance. In comparison, BRISK yielded an average result in all tests while BRIEF yielded a good result in the efficiency and brightness invariance test but has the worst results on scale invariance and rotation invariance tests. Therefore, FREAK achieved the best overall results for mobile AR application using the Mikolajczyk dataset and ALOI dataset, followed by BRISK in 2nd place and ORB and BRIEF in 3^rd^ place respectively.

## Supporting information

S1 File Laboratory Protocol DOI.docx. (DOCX)
